# Current and Emerging Protein Biomarkers for the Diagnosis and Prognosis of Head and Neck Cancer

**DOI:** 10.3390/genes16121493

**Published:** 2025-12-15

**Authors:** Erin Zou, Chethana Venkatraman, Jackson Sweeney, Katy Flannery, Samuel Lailer, Donna Mehdiyar, Komal Parikh, Maryam Salik, Brianna Baughman, Hilal Arnouk

**Affiliations:** 1 Chicago College of Osteopathic Medicine, Midwestern University, Downers Grove, IL 60515, USA; 2 Illinois College of Osteopathic Medicine at The Chicago School, Chicago, IL 60661, USA

**Keywords:** head and neck cancer, oral squamous cell carcinoma, head and neck squamous cell carcinoma, prognostic biomarkers, precision medicine oncology, molecular pathology, targeted treatments, tumor progression, DJ-1, Cornulin

## Abstract

Head and neck cancer represents a heterogeneous group of malignancies. Oral squamous cell carcinoma (OSCC) is the most prevalent form of head and neck cancer, with a rising incidence in recent years. Risk factors for developing OSCC include exposure to carcinogens, such as alcohol and tobacco products, that can lead to molecular alterations in the oral mucosa and progression from premalignant lesions to invasive phenotypes. Despite the relative curative potential of localized OSCC, the overall prognosis of OSCC has not significantly improved for decades due to a frequently delayed diagnosis and limited targeted treatment options. There remains a need to better characterize the molecular biomarkers of OSCC progression, especially in dysplastic mucosal lesions, before their malignant transformation. In this review, we discuss several molecular biomarkers highly implicated in OSCC tumorigenesis that have demonstrated correlation with clinicopathological parameters and clinical outcomes. These biomarkers are typically involved in vital pathways of carcinogenesis, including cell cycle control, growth factor signaling, and stress responses. They include ubiquitous cancer biomarkers such as p53 and PTEN, as well as those more specific to OSCC, such as DJ-1 and Cornulin. Collectively, we envision that a diverse panel of these biomarkers can provide the greatest clinical benefit in enhancing early detection and prognostic accuracy, while some individual biomarkers may also serve as therapeutic targets for personalized approaches to head and neck cancers.

## 1. Introduction

Head and neck squamous cell carcinoma (HNSCC) represents a set of malignancies arising from the mucosal linings of the upper aerodigestive tract. Among these, oral squamous cell carcinoma (OSCC) is the predominant subtype and accounts for around 90% of oral malignancies [1]. The Global Cancer Observatory (GCO) reported more than 389 thousand cases of OSCC and about 188 thousand deaths worldwide in 2022, reflecting an increase in incidence and mortality [2]. The relatively poor prognosis of OSCC is attributable in large part to delayed diagnosis, evidenced by a markedly stratified 5-year survival rate of greater than 80% in localized early disease and less than 30% in advanced distant disease [3].

OSCC develops in the mucosal lining of areas such as the tongue, buccal mucosa, hard and soft palate, and lips. Consumption of tobacco products, alcohol and the human papillomavirus (HPV) are well-established risk factors, with higher incidences of OSCC in males and in Asia [2,4]. Prolonged exposure to numerous carcinogens places the oral mucosa at risk for cumulative genetic and cytological alterations, which can drive the progression from dysplasia to carcinoma in situ to invasive carcinoma. Additionally, the significant heterogeneity of OSCC lesions makes grading and accurate prognosis a challenge. As frequently observed in the oral field cancerization phenomenon, the oral mucosa is at high risk for developing multifocal areas of dysplasia, which can lead to tumor recurrence in multiple sites despite the surgical removal of clinically detectable tumors [5]. This phenomenon highlights the need to identify genetic and proteomic alterations that can serve as biomarkers in tissues that may appear morphologically normal while harboring deleterious mutations. Further, it explains some of the challenges for current treatment strategies and necessitates the use of vigilant surveillance and early detection of recurrent tumors.

**Figure 1:**
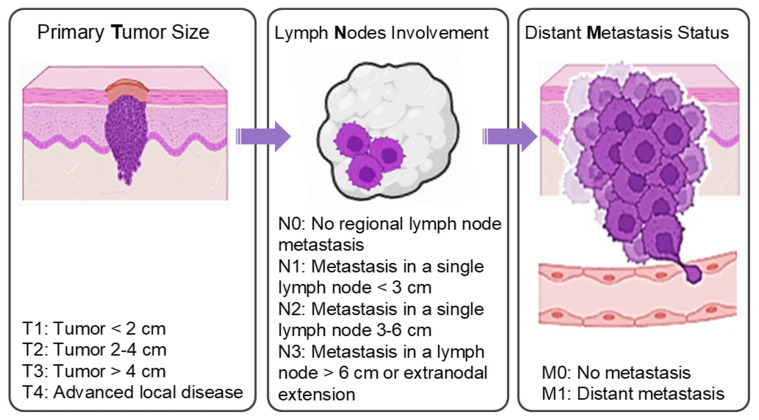
A simplified depiction of the TNM classification system for oral squamous cell carcinoma. T denotes the size of the primary tumor, N indicates the spread of cancer to regional lymph nodes, and the M designation indicates the spread of cancer metastases to distant tissues.

Additionally, current standardized methods for tumor grading for OSCC are still in flux, which further limits accurate diagnosis and prediction of prognosis. Currently, the WHO classification uses a differentiation-based three-tier grading system that continues to underpin diagnostic pathology in head and neck tumors [6]. However, the assigned differentiation grade has repeatedly shown poor correlation with clinical outcomes such as survival rates and therefore provides limited prognostic value [7,8]. On the other hand, TNM clinical staging and the Budding and Depth of invasion (BD) model, which focuses on tumor invasion and aggressiveness, have demonstrated stronger correlations with clinical outcomes and risk stratification to help with decision-making for treatment options [9]. These prognostic models are still being heavily debated and are frequently modified, while their general concepts remain constant [8,10,11] (Figure 1).

Current treatment of nonmetastatic OSCC relies on surgical resection, with the addition of adjuvant therapy using radiation therapy or chemoradiation in cases where there is a high risk of recurrence [12]. However, a significant number of OSCC patients present with advanced disease, where they face a lack of highly effective treatment options leading to poor prognosis [13]. Currently, there is an increased interest in molecular-targeted therapies for HNSCC. For instance, Cetuximab, an anti-EGFR therapy, in combination with radiotherapy, is approved for locally advanced, recurrent or metastatic HNSCC since EGFR overexpression is associated with high local recurrence rates [14,15]. Additionally, the immune-checkpoint PD-1 inhibitor, Pembrolizumab, is approved for recurrent or metastatic HNSCC; and the HPV vaccine has been shown to decrease the incidence of HPV-associated oropharyngeal cancers [16,17]. However, there remains a significant need to identify molecular biomarkers of OSCC to help characterize the different lesions, especially the high-risk premalignant lesions before their malignant transformation when early interventions allow for the most favorable outcomes. This review describes prognostic biomarkers that can classify OSCC lesions into distinct profiles to enhance early detection, accurate risk stratification, and the prediction of malignant transformation in premalignant lesions, ultimately contributing to better clinical outcomes for affected patients.

In this review, we focus mainly on conventional HPV-negative biomarkers that we selected based on key criteria, including biomarkers with high volume of research (substantial evidence and research interest), high prognostic value (multiple studies demonstrating correlation to clinicopathological parameters), and specificity to OSCC. Although many biomarkers are differentially expressed in OSCC, we focused on those with significant correlations with prognostic outcomes such as TNM staging, survival, or recurrence and so demonstrate clinical value. Further, we mainly focus on protein biomarkers that are readily translatable for routine pathology practice using traditional protein expression detection techniques, such as immunohistochemistry.

## 2. Tumor Suppressors and Cell Cycle Biomarkers

### 2.1. p53

The p53 transcription factor, encoded by the *TP53* gene on chromosome 17, is a master cell cycle regulator [18]. TP53 is the most frequently mutated gene in HNSCC, which is observed at higher rates among HPV-negative samples (86%) and in smoking-related HNSCC [19]. Functionally, p53 responds to cellular damage by halting the cell cycle at the G1/S checkpoint, allowing for DNA repair and inducing expression of various genes involved in cellular senescence, apoptosis, and metabolism [20]. However, alterations or inactivation of the wild-type p53 tumor suppressor can occur at various stages of tumor development, promoting genetic instability [21]. *TP53* mutations have been associated with an increased risk of progression from premalignant lesions to invasive carcinoma in OSCC [22]. This pathological progression in the setting of mutated *TP53* can be attributed either to loss of function of the p53 tumor suppressor or gain of function of alternate pathways that drive carcinogenesis [23].

Immunohistochemical (IHC) analysis of OSCC tissue specimens revealed overexpression of p53, although reported positivity rates of p53 expression vary from approximately 28% to as high as 69% in some groups [24,25]. p53 is also valuable as a diagnostic biomarker that can be measured non-invasively or minimally invasively from biological specimens, such as saliva or serum. In unstimulated whole saliva, p53 concentrations are significantly higher in OSCC patients than in healthy controls [26]. Furthermore, p53-related markers are detectable across the premalignant to malignant progression spectrum; both tissue p53 immunoreactivity and circulating serum anti-p53 antibodies occur in oral potentially malignant disorders (OPMDs), such as leukoplakia lesions, and in OSCC. Importantly, the high sensitivity of serum anti-p53 antibodies supports their value as a screening adjunct tool for OPMDs [27].

Several independent studies have demonstrated a significant correlation between p53 expression and the histopathological grade of OSCC, with higher p53 expression often associated with poorly differentiated tumors that display more aggressive biological behavior [25,28,29,30,31,32]. However, other studies have failed to show a significant association between p53 expression and tumor histopathological grade, making it difficult to draw any unifying conclusion about this correlation [33,34,35,36].

In prognostic studies, Yang et al. reported that p53 expression in dysplastic surgical margins of early OSCC was significantly associated with tumor recurrence, indicating poorer relapse-free survival [37]. In a large retrospective cohort study, Solomon et al. demonstrated that p53-positive OSCC had markedly worse survival than p53-negative disease, with an estimated 5-year cumulative survival of about 63% vs. 82%, and 8-year survival of approximately 40% vs. 82%, respectively [38]. These data suggest that p53 expression predicts worse outcomes in OSCC and may potentially aid postoperative risk stratification.

In general, more than half of all human cancers overexpress p53 [20]. Similarly, p53 overexpression is well-documented in oral cancers, underscoring its diagnostic value in OSCC and its potential as an indicator of malignant transformation potential in premalignant lesions.

### 2.2. Cyclin D1

Cyclin D1, also referenced as CCND1, is a member of the Cyclin family and plays a prominent role in regulating cell division, adhesion and invasion in cancer progression [39,40]. Classically, Cyclin D1 functions as a part of the Cyclin D1-CDK4 complex and phosphorylates RB1, which leads to progression through the G1 to the S phase [41]. Additionally, Cyclin D1 is associated with the inhibition of mitochondrial function through the phosphorylation of Nuclear Respiratory Factor 1 (NRF-1) and is involved in regulating transcription factors such as mitochondrial transcription factor A, in sensing and repairing DNA damage, and in inducing chromosomal instability [42,43].

In oral cancer, Cyclin D1 is consistently expressed at higher levels compared to the normal oral mucosa [44,45]. Notably, Cyclin D1 expression progressively increases from oral epithelial precursor lesions to invasive OSCC. This has been demonstrated in IHC studies where Cyclin D1 expression is most frequently associated with oral leukoplakia compared to the normal oral mucosa, and the pRb-/Cyclin D1+ phenotype is associated with transition from premalignant to malignant lesions [44,46]. Further, Cyclin D1 expression was found to be overexpressed in the basal and parabasal layers in non-tumor epithelium adjacent to OSCC [47,48]. These findings support the role of Cyclin D1 expression as a potential indicator of early oncogenic progression and field cancerization in OSCC.

In clinical studies, Cyclin D1 expression significantly correlated with crucial clinicopathological parameters. Cyclin D1 overexpression has been associated with high histological grade, invasive phenotypes, lymph node metastasis, and advanced clinical stages [48,49,50]. Additionally, patients with tongue squamous cell carcinoma, the most common site for OSCC, demonstrated the greatest correlations with Cyclin D1 overexpression and parameters such as nodal status, histopathological grade, and TNM clinical staging, highlighting the utility of Cyclin D1 as a biomarker in this aggressive type of OSCC [51,52,53]. Cyclin D1 overexpression was also associated with poorer clinical outcomes, most notably with decreased overall survival and disease-free survival [50,54,55].

However, therapies targeting Cyclin D1 activity in HNSCC have so far shown limited clinical success. In vitro drug sensitivity studies suggest deregulated Cyclin D1 may contribute to HNSCC chemoresistance, and CDK4/6 inhibitors that inhibit Cyclin D1 activity have shown substantial clinical improvement in breast cancer [56,57,58]. Despite this, CDK4/6 inhibitors have demonstrated minimal effects on survival outcomes in HNSCC or in other solid tumors with Cyclin D-CDK4/6 pathway alterations [59,60]. Cyclin D1 is a promising biomarker for precancerous lesions and HNSCC progression, while further investigation is needed to optimize patient selection and combination regimens with Cyclin D1 inhibitors.

### 2.3. PTEN

**Figure 2:**
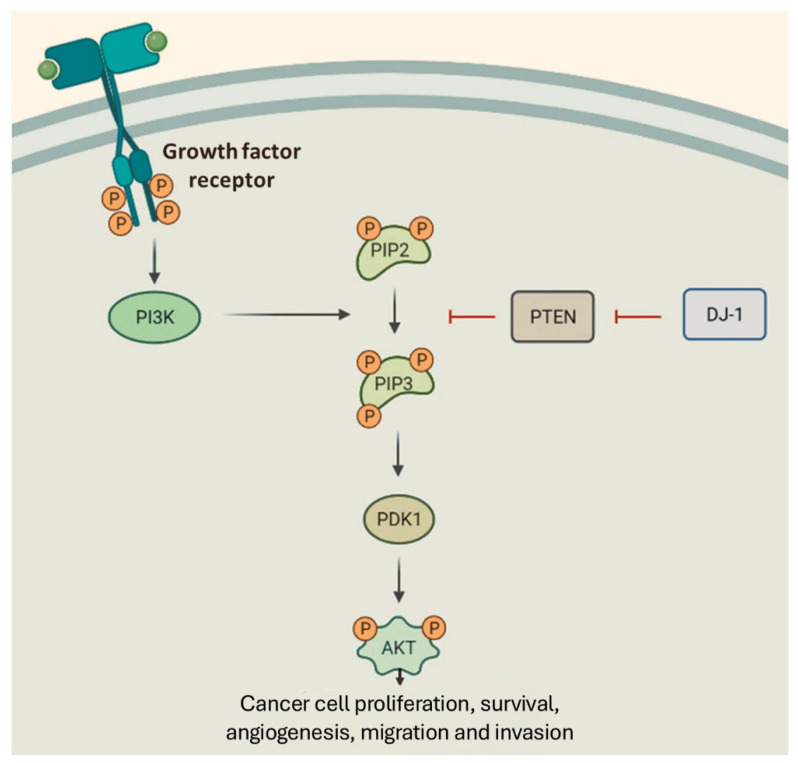
Schematic depiction of the PI3K/Akt signaling pathway that contributes to cancer cell survival, proliferation, invasion, and migration. The role of PTEN and DJ-1 regulatory proteins in modulating the PI3K/Akt signaling pathway is also illustrated.

Phosphatase and Tensin Homolog (PTEN) is a tumor suppressor that normally acts to restrict cell division and metabolism by regulating the PI3K/AKT pathway [61] (Figure 2).

A loss or downregulation of PTEN activity releases this inhibition and promotes uncontrolled cell growth, leading to several types of cancer, including breast, endometrial, ovarian, melanoma, colorectal, and lung cancers. For instance, in gastroesophageal adenocarcinoma (GEA), one-third of the patients with advanced human epidermal growth factor receptor 2 (HER2) GEA demonstrated loss of PTEN, which was associated with significantly lower progression-free survival and overall survival [62]. In melanoma, PTEN expression was downregulated in tumors with thickness greater than 2 mm, which is an indicator of poor clinical outcomes [63].

In HNSCC, PTEN mutations are relatively rare and observed in less than 10% of cases [19,64,65]. However, reduced expression of PTEN is observed in around 30% of HNSCC cases and is an emerging biomarker for its association with tumor progression and its clinical relevance [66,67,68]. In OSCC studies, PTEN expression was significantly decreased in patients compared to healthy controls, with the decrease correlating with progression from dysplastic to malignant phenotypes [67,69]. Moreover, a study combining the administration of a tobacco surrogate in animal models with reduced PTEN expression resulted in the development of oral-specific carcinomas, highlighting the synergistic role of environmental factors and epigenetic PTEN alterations in driving OSCC tumorigenesis [66].

Loss of PTEN activity is also implicated in HNSCC resistance to therapy. In recurrent or metastatic HNSCC treated with Cetuximab, an EGFR inhibitor, low PTEN expression was associated with worse overall survival (hazard ratio 2.27) [68]. In another study, low PTEN expression was detected in 57.3% of patients with oral cavity and oropharynx cancers receiving post-operative radiotherapy, with decreased 5-year loco-regional tumor control compared to high PTEN expression (52.3% vs. 80.9%). However, no significant differences were found in the five-year metastasis-free survival between low and high PTEN expression (81.4% vs. 84.3%) [70]. Additionally, a study using genome editing approaches reported that endogenous and engineered PTEN deletions demonstrated resistance to Cetuximab treatment [71].

PTEN expression may help predict the benefit of therapies in recurrent HNSCC. Loss of PTEN activity is independent of EGFR signaling, with potentially greater prognostic value compared to variables such as regional nodal involvement and EGFR expression [70]. However, there has been limited exploration of PTEN expression in clinical settings in OSCC and its applicability in improving survival outcomes is still uncertain. Based on current studies, PTEN is an emerging biomarker for recurrent HNSCC and using treatments that target the hyperactive downstream pathway (PI3K/AKT/mTOR) in combination with first-line therapies may prove to be uniquely effective in HNSCC patients with decreased PTEN expression.

## 3. Growth Factor Signaling Biomarkers

### 3.1. EGFR

Epidermal growth factor receptor (EGFR) is a widely studied biomarker in HNSCC. EGFR is a transmembrane tyrosine kinase receptor in the ErbB family that binds ligands such as epidermal growth factor (EGF) and transforming growth factor (TGF)-α, triggering dimerization and activation of downstream signaling cascades, notably the RAS–RAF–MEK–ERK and PI3K–AKT pathways [72,73]. Through these signaling pathways, EGFR is involved in cellular proliferation, survival, and differentiation to regulate cell behavior during embryogenesis, tissue regeneration, and bodily homeostasis [74]. Pathologically, EGFR contributes to cancers due to its key role in uncontrolled tumor proliferation. This occurs secondary to EGFR mutations that occur at mutational “hotspots” located in multiple areas of the receptor or its tyrosine kinase domain [75]. When these mutations occur in the keratinocytes of the oral mucosa, EGFR becomes upregulated, making these cells resistant to apoptosis and leading to the development and progression of OSCCs [76].

EGFR expression is frequently increased in OSCC, with an overexpression found in approximately 50–75% of cases [77,78,79]. Based on improved survival data, Cetuximab, an EGFR inhibitor, was approved for locally, regionally advanced, recurrent, and metastatic HNSCC, while development of novel EGFR-targeting therapies and combination regimens is under active investigation [80,81]. In combination trials, Cetuximab and a PD-1 inhibitor have demonstrated improved overall response rates, while combinations with other immunotherapies and small molecular inhibitors are also being tested [82,83,84].

As a prognostic biomarker, high EGFR expression is closely linked to poor overall survival, progression-free survival and increased risk of locoregional relapse [85,86,87,88]. However, EGFR expression does not reliably predict response to anti-EGFR therapies. Emerging evidence suggests that the varied efficacy of EGFR inhibitors could be due to modulation by immune mechanisms and EGFR-specific mutations, limiting the therapeutic advantage [89,90,91,92,93]. For example, patients with tumors that co-expressed EGFR and CD3+ T cells had 5-year survival rates of 68% compared to 40% in those with EGFR overexpression but lacked CD3 infiltration [76]. Overall, EGFR is a promising diagnostic and therapeutic biomarker for OSCC but requires integration with additional biomarkers to better refine risk stratification and response to therapy.

### 3.2. RAS

RAS genes are proto-oncogenes that are frequently implicated in oncogenesis across various tissue types. The Ras protein acts as a molecular switch that, when bound to GTP and activated, triggers numerous downstream effector proteins for cell survival and proliferation [94,95]. Ras mutations result in constitutive activation of these downstream signaling pathways and are found in approximately 19% of patients with cancer and 9% of patients with HNSCC in the United States, underscoring their importance as a biomarker and therapeutic target [96].

The Ras family consists of three members: HRAS (Harvey), KRAS (Kristen) and NRAS (neuroblastoma). While KRAS is responsible for around 75% of cancers with Ras mutations, HRAS has been identified as the dominant isoform mutated in HNSCC and is found in 4–5% of cases [96,97,98,99]. However, variable findings exist where the dominant isoform and incidence of mutated HRAS differ based on ethnicity and study population [100,101,102]. More generally, studies have shown HRAS expression to be increased in OSCC. IHC analyses demonstrate increased HRAS expression in OSCC and oral epithelial precursor lesions compared to the normal oral mucosa, while increased HRAS immunoreactivity correlated with a positive M status, indicating the presence of distant metastases, and inversely with survival rates [101,103].

Clinically, oncogenic HRAS has emerged as a significant therapeutic target and may underlie treatment resistance in HNSCC. This is evidenced in studies where KRAS/HRAS mutations have been associated with poor progression-free survival in the first-line recurrent setting with Cetuximab, while HRAS depletion in cells with oncogenic HRAS restored sensitivity to growth inhibition by Cetuximab [104,105]. Additionally, the Gly12Ala HRAS mutation was associated with approximately 66.6% of recurrent OSCC cases compared to 10% of OSCC primary cases [106].

Tipifarnib is a farnesyltransferase inhibitor that specifically inactivates HRAS out of the three Ras proteins [107]. In a clinical trial, patients with recurrent or metastatic HNSCC and mutant HRAS, Tipifarnib treatment resulted in an objective response rate of 55% and a median overall survival of 15.4 months [108]. Consequently, Tipifarnib was granted Breakthrough Therapy Designation by the U.S. Food and Drug Administration (FDA) for the treatment of patients with recurrent or metastatic HRAS mutant HNSCC and is currently being studied as a combination therapy with a PI3K inhibitor [109].

Overall, there are significant challenges for patients with HRAS-mutant HNSCC, where there are high rates of recurrence (50–67% relapse in less than 6 months) and short disease-free survival (4 months) [98]. Tipifarnib is a promising additive treatment and HRAS is an important biomarker in targeting this patient population.

### 3.3. JAK/STAT Pathway

The JAK/STAT signaling axis consists of four Janus Kinase proteins (JAK1, JAK2, JAK3, and TYK2) and seven Signal Transducer and Activator of Transcription proteins (STAT1-4, STAT5A, STAT5B, and STAT6). These proteins mediate signal transduction from cytokine and growth factor receptors to the nucleus, regulating gene expression [110]. Under homeostatic conditions, STAT proteins are activated by phosphorylation, often by a member of the JAK family, before dimerizing, relocating to the nucleus, and exerting their effects by influencing gene expression [111]. Dysregulation of the JAK/STAT pathway is frequently found in several cancers, and its hyperactivation is implicated in oncogenesis by promoting proliferation, survival, immune evasion, and therapeutic resistance [112]. This dysregulation is a common feature of HNSCC and highlights members of the JAK/STAT pathway as potential biomarkers and treatment targets [113].

Most studies investigating the JAK/STAT pathway in HNSCC have focused on STAT3. Constitutive activation of STAT3 is frequently observed in HNSCC and is associated with poor prognosis and resistance to therapy. STAT3 activation has shown approximately an 11-fold increase in tumor specimens and a 9-fold increase in histologically normal mucosa surrounding tumors in HNSCC patients, when compared with non-cancer controls [113]. HNSCC cells with higher levels of phosphorylated STAT3 also promoted faster proliferation, while mechanisms linking STAT3 overactivation and HNSCC, such as via RTPRT promoter methylation and upregulated TGF-β, are under investigation [114,115,116]. STAT3 overexpression has also been consistently linked to poor prognostic outcomes such as decreased survival rates [117,118].

Importantly, upregulation of STAT3 may contribute to treatment resistance. STAT3 signaling was found to be upregulated after anti-EGFR therapy in HNSCC patients, while inhibition of STAT3 signaling restored sensitivity to therapy in in vitro and animal models [119]. Translational studies demonstrated that adding STAT3 inhibitors to anti-EGFR therapies significantly enhanced antitumor effects [120]. As a therapeutic mechanism, JAK/STAT inhibition has shown promise in preclinical models. AZD1480, a potent JAK2 inhibitor, significantly reduced HNSCC cell proliferation in vitro and suppressed tumor growth in heterotopic xenograft models [110]. Similarly, platinum-based chemotherapeutic agents were found to interact with the SH2 domains of multiple STAT proteins, promoting their dephosphorylation and downregulating pro-survival genes [121]. Recent studies have also reported on the potential of other members of the JAK/STAT family to be molecular biomarkers and therapeutic targets for HNSCC. STAT1 may also serve as a marker of radiation therapy resistance, and targeting STAT1 could enhance T-cell-mediated immune activation [122]. Meanwhile, TYK2 overexpression was associated with improved survival outcomes and increased immune cell infiltration, serving as a positive prognostic indicator for HNSCC [123].

Overall, the clinical efficacy of JAK/STAT inhibition is limited and remains to be fully established in larger trials. However, scientific evidence shows that the JAK/STAT signaling cascade is an important mediator of HNSCC pathogenesis and treatment resistance, thus, warrants further investigation for its prognostic and therapeutic applications.

## 4. Stress Response and Cellular Protection

### 4.1. DJ-1

DJ-1, also known as Parkinson’s disease protein 7 (PARK7), is an emerging oncogenic biomarker with high expression levels reported in various types of cancer, including breast, pancreatic, and supraglottic cancers [124,125,126]. DJ-1 plays a critical role in promoting cell survival through modulation of key intracellular signaling pathways. Specifically, DJ-1 has been shown to inhibit the expression of PTEN, a well-recognized tumor suppressor, thereby enhancing the activation of the PI3K/AKT pathway and promoting cancer cell survival and proliferation [127]. Through this mechanism, DJ-1 upregulation contributes to tumorigenesis, metastatic potential, and poorer clinical outcomes across multiple cancer types [128]. Notably, DJ-1 downregulation has also been shown to induce cellular differentiation and suppress proliferation, further supporting its role as an oncogenic driver [129].

**Figure 3:**
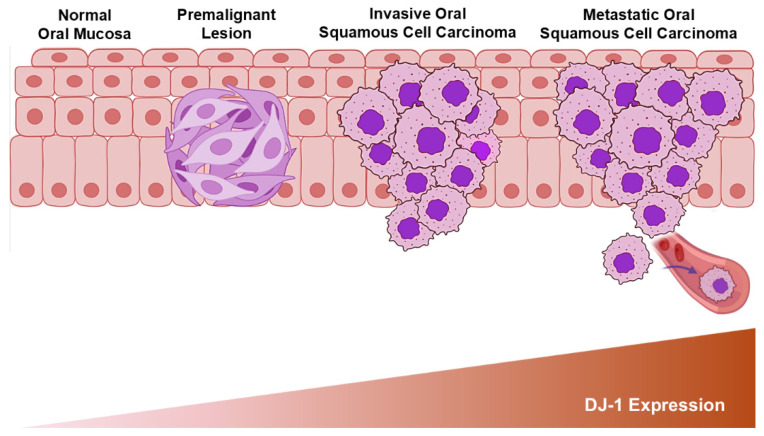
Schematic graph showing the correlation between the upregulation in DJ-1 expression and the progression of squamous cell carcinomas from dysplastic premalignant lesions to invasive and metastatic phenotypes.

In OSCC, DJ-1 is a potential biomarker for disease progression, cellular differentiation, and histopathological grading. DJ-1 expression increases in a stepwise fashion during OSCC progression, with the metastatic phenotype showing a four-fold increase in expression compared to normal gingival keratinocytes. Similarly, metastatic OSCC cells display a 2.3-fold and 1.8-fold increase in DJ-1 expression compared to early dysplastic oral keratinocytes and locally invasive OSCC, respectively [67] (Figure 3).

Additional studies have shown increased DJ-1 expression in leukoplakia, a common precancerous lesion to OSCC, at levels greater than normal oral mucosa but less than malignant OSCC lesions [129].

Furthermore, DJ-1 expression has been found to correlate with the histopathological tumor grade, with higher expression associated with poorly differentiated OSCC lesions and a more aggressive biological behavior. Compared to the normal oral mucosa, OSCC demonstrates 3-fold greater DJ-1 immunoreactivity. With OSCC biopsies, greater DJ-1 immunoreactivity was observed in the tissue samples with high histopathological grades (G2, G3) [130].

Collectively, DJ-1 is a promising biomarker for the early detection of oral cancer. The progressive increase in DJ-1 expression provides great prognostic value and may help determine the histopathological grade and stratification of treatment options.

### 4.2. Cornulin

Cornulin, encoded by the *CRNN* gene, has recently gained attention for its potential role in squamous cell carcinomas [131]. Although its precise molecular mechanisms remain to be fully elucidated, studies have suggested that Cornulin functions as a tumor suppressor, particularly by arresting the cell cycle at the G1/S transition [132]. Transfection of malignant cell lines to overexpress Cornulin has been shown to significantly reduce cellular proliferation by halting progression through the G1/S checkpoint [133]. Notably, downregulation of Cornulin has been consistently reported in the squamous cell carcinomas of the cervix, esophagus, and head and neck [132,134,135].

In OSCC, Cornulin expression is markedly diminished. Quantitative analysis of OSCC cell lines representing different stages of disease progression revealed a 3-fold and 24-fold downregulation of Cornulin expression in dysplastic oral keratinocytes and locally invasive OSCC cells, respectively, compared to normal oral keratinocytes [136]. In a proteomic analysis evaluating 500 proteins across the OSCC spectrum, Cornulin emerged as the most significantly downregulated protein, demonstrating a consistent stepwise decline in expression from normal mucosa through dysplasia and invasive carcinoma. Intriguingly, the same study showed that Cornulin levels were also decreased in the saliva of OSCC patients, highlighting its potential as an accessible biomarker that can be measured using a non-invasive collection of saliva samples [137]. While no recurrent mutations have been identified in the *CRNN* gene to explain its downregulation, Cornulin loss has been associated with microsatellite instability (MSI)-positive OSCC cell lines and poorer prognosis [138].

IHC analysis has corroborated these findings. Cornulin staining intensity was highest in normal oral mucosa and progressively decreased with OSCC progression, including in premalignant leukoplakia lesions. Notably, IHC was able to differentiate between high-grade and low-grade premalignant lesions, suggesting a potential role for Cornulin in early detection and risk stratification [139].

Beyond its diagnostic applications, Cornulin may also serve as a predictive biomarker for local relapse, a major clinical challenge in OSCC management. Despite clear histological margins, approximately 10–30% of OSCC patients experience local recurrence post-surgery [140]. A study using IHC analysis found that Cornulin downregulation in surgical margins was associated with a 34-fold increased risk of local relapse in comparison to surgical margins displaying normal levels of Cornulin expression [141]. Incorporating Cornulin expression analysis into surgical pathology could enhance margin assessment and potentially improve prognosis by identifying patients at greater risk of recurrence.

## 5. Invasion and Angiogenesis Biomarkers

### 5.1. VEGF

The importance of Vascular Endothelial Growth Factor (VEGF) has long been recognized in cancer biology as a central regulator of angiogenesis, which is the formation of new blood vessels from pre-existing vasculature. While VEGF is essential for normal tissue growth and repair, its overexpression has been strongly implicated in the pathogenesis and progression of solid tumors, including OSCC [142]. Early investigations established a clear association between increasing vascularity and the progression from normal oral mucosa to dysplasia and ultimately to invasive squamous cell carcinoma [143]. Subsequent studies expanded VEGF’s oncogenic role, revealing its contribution to the tumor microenvironment through suppression of anti-tumor immune responses and enhancement of tumor cell survival and proliferation [144]. Collectively, these findings have positioned VEGF as a critical driver of OSCC progression and a candidate biomarker for disease monitoring.

IHC has been used extensively to quantify VEGF expression in OSCC tissue. Mineta et al. reported that 45% of OSCC samples demonstrated high VEGF expression, which correlated significantly with increased lymph node metastasis and poorer clinical outcomes [145]. Similarly, another study found that strong VEGF staining in tumor samples correlated with a 1.88-fold higher risk of death within two years compared to tumors with weak or negative staining [146]. Additionally, VEGF expression has been linked to tumor size; one study reported that 14 of 17 tumors larger than 8 cm^3^ exhibited moderate to high VEGF staining, although this expression was found to be independent of tumor grade [147]. More recently, Ilango et al. demonstrated a stepwise increase in VEGF positivity across disease stages, with 2% of normal mucosa, 66% of dysplastic lesions, and 100% of OSCC tissues showing detectable VEGF staining, thereby further highlighting its role in tumor initiation and progression [148].

In addition to tissue analysis, several studies have examined VEGF levels in the blood of OSCC patients. Siemert et al. reported that pre-treatment plasma VEGF concentrations above 26 ng/L were significantly associated with poorer prognosis and reduced event-free survival [149]. Another study demonstrated approximately a 37-fold increase in VEGF-A mRNA expression in whole blood samples from OSCC patients compared to healthy individuals [150]. In parallel, Xu et al. demonstrated that circulating VEGF concentrations exceeding 130.4 pg/mL not only differentiated OSCC patients from controls but also served as independent prognostic indicators for both overall and progression-free survival rates [151].

Given the strong evidence linking high VEGF expression in HNSCC to poor prognosis, multiple anti-VEGF agents are being investigated in recurrent and metastatic cases, and often as combination therapies. Notably, Bevacizumab is a monoclonal antibody targeting VEGF and leading to improved objective response rate and progression-free survival, but showed no improvements in overall survival and increased toxicity events such as bleeding [152]. More recently, Ramucirumab, a selective VEGFR2 inhibitor, was studied in combination with anti-PD-1 immune checkpoint inhibitor, Pembrolizumab, in clinical trials and reported favorable safety and objective response rates [153].

Taken together, the evidence underscores VEGF’s central role in the initiation, progression, and clinical course of OSCC. Whether measured through tissue-based IHC or circulating levels in the blood, elevated VEGF expression consistently correlates with tumor aggressiveness, lymph node metastasis, and poorer survival rates. These findings not only validate VEGF as a promising biomarker for disease monitoring but also highlight its potential utility in guiding targeted therapeutic strategies aimed at disrupting tumor angiogenesis and improving patient outcomes in OSCC.

### 5.2. Matrix Metalloproteinases

Matrix metalloproteinases (MMPs) are a family of zinc-dependent endopeptidases responsible for the degradation and remodeling of the extracellular matrix (ECM), playing critical roles in normal physiological processes such as wound healing, angiogenesis, and tissue regeneration. Among these, MMP2 and MMP9, also known as gelatinases A and B, respectively, hold significant relevance in cancer biology due to their ability to degrade type IV collagen, a major structural component of basement membranes [154]. This function is especially critical in the context of tumor invasion and metastasis. Consistent with their biological roles, MMP2 and MMP9 break down ECM to facilitate the local invasion and lymphovascular spread of OSCC [155]. Given their biological roles, MMP2 and MMP9 have been extensively investigated as potential biomarkers of OSCC aggressiveness, histopathological grade, and clinical outcome.

Multiple studies have demonstrated a strong correlation between MMP9 expression and increasing tumor grade and stage. Patil et al. reported that the mean IHC expression score of MMP9 increased from 2 in stage I tumors to 3.4 in stages III and IV [156]. Similarly, Atla et al. observed a statistically significant rise in MMP9 expression in the less differentiated, and more aggressive OSCC tumors, with high expression detected in 20 of 35 moderately differentiated tumors compared to only 2 of 30 well-differentiated cases. MMP2 expression has shown comparable associations [155]. In a study by Lawal et al., 100% of poorly differentiated OSCC samples exhibited high MMP2 expression, compared to 40% of well-differentiated and 23% of moderately differentiated tumors [157].

In addition to the histopathological grade, MMP2 and MMP9 expression levels have been linked to lymph node metastasis and survival outcomes. Patil et al. reported that tumors without nodal invasion had a mean MMP9 immunoreactivity score of approximately 2, whereas those with regional or distant metastases demonstrated significantly higher expression, averaging a score of 3.5 [156]. High MMP2 expression has also been shown to correlate with poor prognosis. Hoffman et al. reported that MMP2 was an independent prognostic marker in OSCC, with elevated expression associated with reduced survival rates [158].

These findings underscore the pivotal roles of MMP2 and MMP9 in OSCC pathogenesis. By enabling extracellular matrix degradation and facilitating tumor invasion, their overexpression contributes directly to disease progression and metastasis. As a therapy, MMP inhibitors, such as Marimastat, demonstrated promising preclinical data in HNSCC, but showed no significant clinical benefit and dose-limiting musculoskeletal adverse effects in clinical trials of several solid tumors [159,160,161]. Ultimately, while their therapeutic potential is still being explored, MMPs have consistently correlated with tumor grade, advanced stage, and poorer prognosis, which highlights their potential as clinically relevant biomarkers for OSCC.

### 5.3. CD44

Cluster of Differentiation 44 (CD44) is a cell surface glycoprotein that is a recognized marker for cancer stem cells (CSC), a subpopulation of tumor cells that is capable of self-renewal and is associated with carcinogenesis, tumor progression, metastatic spread, and resistance to chemotherapy and radiation therapy [162]. CD44 functions mainly as a receptor for hyaluronic acid, a major component of the extracellular matrix, and is expressed in a wide range of cell types for its roles in cellular structure and communication. Through alternative splicing, the human CD44 gene consists of the standard (CD44s) and variant (CD44vv) isoforms that are implicated in diverse oncogenic processes from angiogenesis, migration, invasion, to metastasis [163,164].

CD44 expression is frequently upregulated in OSCC. Analysis of TCGA data reported that HNSCC has the second-highest CD44 expression among all cancer types [165]. Further studies correlate high CD44 expression with OSCC and oral dysplastic lesions [166,167,168,169,170]. CD44 overexpression is also consistently associated with numerous clinicopathological parameters, including the TNM clinical staging. Specifically, CD44 was found to be overexpressed in 54.7% of TNM stage IV samples compared to only 5.7% in TNM stage I [171]. Notably, CD44 expression consistently predicts lymph node metastasis, highlighting CD44’s role as a marker of tumor invasion and aggressiveness [166,169,171,172].

High CD44 expression was also found to independently predict worse overall, disease-specific, and disease-free survival rates in OSCC [166,172,173,174]. However, there have been variable findings with some studies demonstrating no significant associations with survival rates and clinical outcomes [167,170]. These discrepancies could potentially be attributed to small sample sizes and the role of different CD44 isoforms in cancer progression and behavior. Importantly, the expression of CD44 variants has been implicated in chemoresistance [175,176]. Overall, dysregulation of CD44 is strongly linked to tumorigenesis and several clinicopathological parameters. Thus, it may serve as a biomarker of OSCC aggressiveness and metastatic spread. Although the translation of CD44 into a therapeutic target is not fully developed, several monoclonal antibodies, chimeric antigen receptor-engineered natural killer cell (CAR-NK) therapy, and small molecular inhibitors are currently being explored in preclinical and clinical trials [177,178,179,180,181]. Further studies are needed to stratify the risk and therapies associated with the different CD44 isoforms.

## 6. Differentiation and Stem Cell Regulation Biomarkers

### NOTCH1

The NOTCH1 protein plays a crucial role in regulating cell proliferation, differentiation, and apoptosis across various tissue types. Its biological behavior is highly context-dependent as it can act either as a tumor suppressor or as an oncogene depending on the tumor type [182,183,184]. However, its precise function in the pathogenesis of oral cancer remains controversial, with different studies presenting evidence for both oncogenic and tumor-suppressive roles [185].

It has been shown that NOTCH1 expression increases during the transformation from normal oral mucosa into OSCC [184,186]. Similarly, Ding et al. reported that NOTCH1 expression rises in premalignant leukoplakia lesions and correlates with the severity of epithelial dysplasia [187]. Moreover, activation of the broader NOTCH signaling cascade has been observed in HNSCC, with upregulation of the receptor ligands such as JAG1, JAG2, and NUMB in tumor tissue compared to normal mucosa [186].

Additionally, the expression of NOTCH1 appears to vary according to tumor differentiation status. Mohammedsaleh et al. demonstrated that poorly differentiated OSCC samples exhibit reduced NOTCH1 expression compared to well-differentiated lesions, suggesting loss of NOTCH1 signaling in the more aggressive tumors [188]. On the other hand, Upadhyay et al. found that elevated NOTCH1 expression correlated with lymph node metastasis in tongue OSCC, indicating a potential association between NOTCH1 upregulation and tumor spread [189].

Studies correlating NOTCH1 expression with clinical outcomes have somewhat conflicting findings. Most studies report that increased NOTCH1 expression correlates with poor prognosis and decreased survival outcomes [188,189,190,191]. However, Kaka et al. observed that NOTCH1 overexpression was associated with improved survival in oropharyngeal carcinoma, underscoring the context-dependent nature of its biological role [192].

Current evidence highlights the complex role of NOTCH1 in the carcinogenesis of HNSCC. While upregulation of NOTCH1 and its associated pathway components is commonly observed during the progression from normal mucosa to carcinoma, its influence on tumor differentiation, metastasis, and prognosis appears to vary across anatomical sites and tumor subtypes. Despite the complexity of its role, the accumulating evidence suggests that NOTCH1 holds considerable potential as an early molecular biomarker for the diagnosis and prognosis of HNSCC.

## 7. HPV and Other Relevant Molecular Biomarkers

While this review primarily focuses on non-HPV-associated protein biomarkers of OSCC, there are several thoroughly researched biomarkers that warrant brief mention due to their ongoing clinical relevance. In particular, the HPV status, cyclin-dependent kinase inhibitor 2A (p16), retinoblastoma protein (pRb), programmed cell death protein 1 (PD-1), human telomerase reverse transcriptase (hTERT), and long non-coding RNAs (lncRNAs) and microRNAs (miRNAs) have all been implicated in OSCC pathogenesis. They remain important to acknowledge, given their established and evolving roles in both HPV-positive and HPV-negative head and neck cancer phenotypes.

### 7.1. Human Papillomavirus (HPV)

HPV status has emerged as a key biomarker in HNSCC, but its clinical relevance differs significantly depending on the tumor’s anatomical location. It is most strongly associated with oropharyngeal squamous cell carcinoma (OPSCC), particularly tumors of the tonsils, base of tongue, and soft palate. In contrast, HPV plays a limited role in squamous cell carcinoma of the oral cavity, which arises in sites such as the anterior tongue, floor of mouth, buccal mucosa, hard palate, and alveolar ridge. HPV-positive OSCC tends to occur in younger, non-smoking patients and is associated with a more favorable prognosis and response to chemoradiation therapy. On the other hand, HPV-negative OSCC is often linked to traditional risk factors such as tobacco and alcohol use, with worse outcomes [193]. Overall, only 6% of OSCC cases are HPV-positive, and it is thought that HPV is unlikely to play a major oncogenic role in most oral cavity tumors [194]. HPV-associated OSCC has instead been described as an uncommon but distinct subtype, typically showing non-keratinizing histology, basaloid features, and strong, diffuse p16 expression [195]. Recognizing these distinct features can be useful diagnostically.

Diagnostic evaluation of HPV status typically begins with p16 immunohistochemistry, followed by HPV DNA PCR, or RNA in situ hybridization, to confirm viral activity. Although routine HPV testing is a standard test for oropharyngeal tumors, its role in OSCC remains unclear. More studies are needed to clarify the prognostic and therapeutic implications of HPV in cancers occurring in the oral cavity.

### 7.2. p16

p16 is a tumor suppressor protein encoded by the CDKN2A gene. Its overexpression serves as a marker of HPV-associated carcinogenesis. This overexpression of p16 in HPV-positive OSCC is driven by E7-mediated degradation of the pRb, which leads to uninhibited p16 transcription [196]. Patients with OSCC tumors that are positive for p16 tend to have better overall and cancer-specific survival rates [197]. IHC analysis reveals that strong, diffuse nuclear and cytoplasmic p16 staining is typically found in HPV-driven OSCC, whereas p16 expression is often heterogeneous or absent in HPV-negative tumors [198]. p16 expression has been investigated as a predictive biomarker for response to therapy. Several studies have demonstrated that p16-positive tumors are more responsive to radiotherapy and chemoradiation, likely due to their distinct molecular and immune profiles [199]. P16-positive tumors combine tumor-intrinsic defects in double-strand-break repair and lower genomic instability with a pre-existing, highly inflammatory immune microenvironment. These features increase sensitivity to DNA-damaging therapy and promote radiation-induced, immune-mediated tumor control [200]. While p16 is not currently used to guide therapy in HPV-negative OSCC, its expression may still contribute to tumor characterization as part of a broader biomarker panel.

### 7.3. Retinoblastoma Protein (pRb)

pRb is another key effector molecule in the p16/Cyclin D1/CDK4/6/Rb pathway which controls G1/S transition by regulating E2F activity [201]. pRb is a tumor suppressor involved in numerous vital processes, including cell cycle control, replication, genetic stability, and apoptosis, and is thereby implicated in the pathogenesis of numerous malignant neoplasms, including head and neck cancers [202,203]. Given that HPV directly inactivates pRb, the loss of pRb has been significantly associated with HNSCC cases and associated premalignant lesions [44,204,205]. Accordingly, pRb expression was found to inversely correlate with p16 expression, where pRb-positive samples were found to be p16-negative and vice versa [205,206]. High p16/ low pRb expression defines a specific subset of HPV-associated OSCC that is associated with improved survival [204]. However, several prognostic studies of independent pRb expression generally demonstrate mixed results, with variable to no correlation with survival outcomes and largely no correlation with clinicopathological parameters [206,207,208,209]. Other studies have linked pRb overexpression with OSCC progression and clinicopathological parameters [210,211]. These variable findings may be attributed to the differential influence of HPV infection, where HPV-positive oncogenesis is associated with E6/E7 oncoproteins, inactivation of p53 and pRB, and overexpression of p16, while HPV-negative OSCC is associated with more frequent p53 mutations, loss of p16, and presence or overexpression of pRb [204,212,213,214,215]. In summary, pRb expression is highly associated with HPV-positive OSCC but currently has limited prognostic implications. For HPV-negative OSCC, pRb expression should be best interpreted within the broader context of the p16/Cyclin D1/Rb pathway when being considered as a biomarker.

### 7.4. Programmed Cell Death Protein 1 (PD-1)

Programmed cell death protein 1 (PD-1) is an immune checkpoint receptor that is expressed on activated T cells. It plays a critical role in inhibiting anti-tumor immune responses. Engagement of PD-1 with its ligand PD-L1, which is frequently upregulated on the surface of cancer cells, including OSCC cells, and stromal elements, suppresses cytotoxic T-cell activity and promotes T-cell exhaustion, enabling tumor immune evasion and escape [216].

Immunohistochemical and multiplexed analyses in OSCC demonstrated that PD-1 is commonly expressed on tumor-infiltrating lymphocytes and that PD-L1 is frequently, but variably, expressed on OSCC tumor cells and tumor stromal cells. Several studies report high rates of PD-1+ tumor-infiltrating lymphocytes and PD-L1-positive tumors in OSCC, though prevalence varies by patients’ cohort and scoring method [217,218,219]. Mechanistic studies support a functional role for PD-L1 in driving tumor growth and inhibiting anti-tumor immunity in head and neck cancers, which further reinforces the clinical relevance of these biological findings [220].

Clinical trials have established that the administration of immune checkpoint inhibitors targeting PD-1, such as Pembrolizumab and Nivolumab, significantly improves survival in recurrent or metastatic HNSCC regardless of the HPV status [16,221]. PD-L1 expression, Tumor Mutational Burden (TMB), and immune cell infiltration have all shown potential as predictive biomarkers for the response to immunotherapy in OSCC. However, their interpretation remains somewhat limited by substantial variability in detection assays, differences in scoring protocols, including tumor-cell vs. immune-cell scoring, PD-L1 protein Tumor Proportion Score (TPS) vs. Combined Positive Score (CPS), and inconsistent thresholds for defining positivity, which hinders reliable comparison across the different studies [219,222].

Prognostic findings for PD-1/PD-L1 in OSCC remain somewhat variable. Some studies link high tumor-cell PD-L1 expression to worse outcomes, while others find that PD-L1 expression on immune cells or the presence of dense CD8+ TILs correlates with better survival, which highlights the context-dependent nature of this pathway [218,223]. Emerging evidence also points to the tumor-draining lymph node microenvironment, including PD-L1–positive dendritic cells and exhausted PD-1–positive CD8+ T cells, as contributors to recurrence and nodal spread, suggesting that the prognostic utility may extend beyond the primary tumor [217,222].

Overall, PD-1 and PD-L1 are biologically and clinically meaningful biomarkers in OSCC, reflecting tumor–immune interactions and predicting response to PD-1 blockade in recurrent or metastatic disease. However, their broader use for prognosis or patient selection is currently limited by inconsistent detection methods, variable correlations across studies, and the limited OSCC-specific prospective trials. Standardized assays, defined scoring thresholds, and integrated evaluation of tumor, immune, and lymph-node compartments will be essential to solidify their diagnostic, prognostic, and predictive roles in oral cancer.

### 7.5. Human Telomerase Reverse Transcriptase (hTERT)

hTERT is the catalytic subunit of telomerase and is responsible for maintaining telomere length and enabling replicative immortality. In normal somatic cells, telomerase activity is typically repressed, but hTERT is frequently reactivated in malignant tumors, including OSCC. This reactivation promotes unlimited cancer cell divisions and tumor progression. Elevated hTERT expression has been observed in head and neck cancers, where it correlates with increased cell proliferation, tumor invasiveness, and poor clinical outcomes. hTERT expression was significantly higher in oral and salivary gland carcinomas compared to the normal counterpart tissues [224]. Additionally, hTERT overexpression has been significantly associated with decreased overall survival and adverse prognostic features [225]. These findings suggest hTERT as a promising biomarker for predicting disease outcomes and may also serve as a potential target for developing future OSCC therapies.

### 7.6. Long Non-Coding RNAs (lncRNAs) and microRNAs (miRNAs)

LncRNAs and microRNAs are promising molecular biomarkers for OSCC with substantial evidence supporting their clinical relevance. LncRNA has diverse functional roles from modulating tumor invasion to immune cell infiltration, and its increased expression is associated with poor prognosis and high diagnostic accuracy [226,227]. Additionally, several microRNA candidates have been identified that distinguish OSCC patients from healthy controls with high specificity and sensitivity and predict disease recurrence and survival [228,229,230]. miRNAs are also stable and detectable in serum, lending excellent utility as non-invasive biomarkers for early detection and monitoring disease progression [228].

In summary, HPV status, p16, pRb, PD-1, hTERT, lncRNAs and miRNAs represent a diverse group of biomarkers with implications spanning diagnosis, prognosis, and therapeutic response in OSCC. While these markers are not exclusive to oral cavity tumors, their presence—particularly in specific molecular or anatomic subgroups—may aid in refining risk stratification and guiding treatment decisions. Further investigation into their role in non-oropharyngeal OSCC is warranted to establish their prognostic significance and utility in clinical practice.

## 8. Conclusions

In this review, we provide an overview of select biomarkers with evidence supporting their unique expression patterns in HNSCC. While most of the relevant literature focuses on biomarker expression in the advanced stages of cancer, we also emphasize biomarkers altered at the earlier stages of dysplastic premalignant lesions, and those that have demonstrated strong correlation with clinical outcomes parameters such as survival or relapse rates. Taken together, these biomarkers may grant the most diagnostic and therapeutic advantages: first, by allowing earlier detection of these cancers when targeted treatments are most successful and second, by serving as therapeutic targets themselves.

The biomarkers we discussed cover a range of pathways involved in tumor development, reflecting the heterogeneous progression schemes of OSCC from dysplasia to advanced carcinoma lesions. Notable tumor suppressors, such as p53, play a central role in cell cycle control and are frequently altered in cancer. For instance, 86% of HPV-negative HNSCCs have mutations in TP53 [19]. On the other hand, the EGFR oncogene is frequently amplified in HNSCC and contributes to uncontrolled cell proliferation [19,78]. Meanwhile, recently discovered biomarkers like DJ-1 and Cornulin play important roles in cellular stress response and have unique expression patterns in OSCC, allowing them to serve as biomarkers for OSCC progression [130,131,136].

Furthermore, we highlighted biomarkers that have well-established correlations with histopathological grading and clinical staging and have demonstrated clinical translatability. In the reported studies, the most frequently measured clinical outcomes were survival rates and tumor recurrence, with additional studies on therapeutic effectiveness with therapies targeting biomarkers such as EGFR and HRAS. Important clinical outcome trends emerged, including the independent association between lower overall survival rates and increased expression of DJ-1, VEGF, MMP-2, or Cyclin D1, and between lower progression-free survival and increased VEGF expression [54,128,145,146,158]. Studies documenting relapse rates reported correlations between lower disease-free survival and increased Cyclin D1 or VEGF expression, and between increased tumor recurrence and decreased Cornulin expression or increased HRAS expression [52,55,106,141]. Additionally, increased expression of PTEN was associated with improved loco-regional control (LRC).

Overall, continuing research in OSCC will allow us to further categorize biomarkers by their prognostic significance. For example, Cyclin D1, PTEN, and Cornulin serve as markers of transformation with a consistent and predictable pattern of expression from dysplasia to OSCC [46,67,69,136]; Cyclin D1, DJ-1, and NOTCH1 are early markers found in pre-malignant epithelium [47,48,129,186]; and PTEN, HRAS, and STAT3 are markers of treatment resistance with potential as therapeutic targets in combination therapies [68,70,104,105,119,120]. Other biomarkers, VEGF, MMPs and CD44, have strong prognostic value but demonstrate modest clinical translatability, while EGFR has limited prognostic value but is a promising target as a first-line treatment for advanced HNSCC. These biomarkers provide diverse but synergistic utility in detecting and managing head and neck cancers.

## 9. Future Perspectives

Ultimately, integrating biomarkers into current diagnostic and prognostic models, such as TNM staging or histopathological grading, may provide the greatest clinical value. Although molecular biomarkers are currently not considered the standard of care for the primary diagnosis of OSCC, they may provide enormous benefit for early detection and risk stratification, particularly in patients with pre-malignant lesions or in high-risk patient groups (i.e., tobacco use, alcohol use, HPV infections). Further, these biomarkers fulfill diverse prognostic functions—serving as markers of early malignant transformation in premalignant lesions, recurrence, or treatment resistance—and together may provide synergistic diagnostic benefit. However, given the molecular heterogeneity of OSCC, a single biomarker is unlikely to capture adequate insights into the different tumor progression schemes. Instead, we envision the use of a panel of biomarkers, with likely candidates detailed in this article, which cover numerous pathways and a variety of tumor behavior characteristics.

Importantly, a diverse biomarker panel can reflect the heterogeneity of OSCC and guide a multimodal, personalized approach to treatment. A promising example is the successful use of PD-L1 expression to guide immunotherapy, while EGFR and HRAS are emerging therapeutic biomarkers that still need to consistently demonstrate significant clinical benefits before becoming a standard of care [108,221,231]. Overall, only few biomarkers are currently being used to guide targeted therapy selection despite our significantly improved understanding of the molecular drivers of OSCC carcinogenesis and the limitations of current treatments due to acquired resistance [232,233]. Ultimately, biomarkers can enhance diagnostic sensitivity and specificity and allow clinicians to better stratify the risk and personalize treatment plans for patients with head and neck cancer.

## Data Availability

No new data were created or analyzed in this study.
